# Transcriptome profiling of monocytes from XLA patients revealed the innate immune function dysregulation due to the *BTK* gene expression deficiency

**DOI:** 10.1038/s41598-017-06342-5

**Published:** 2017-07-28

**Authors:** Hoda Mirsafian, Adiratna Mat Ripen, Wai-Mun Leong, Chai Teng Chear, Saharuddin Bin Mohamad, Amir Feisal Merican

**Affiliations:** 1 Institute of Biological Sciences, Faculty of Science, University of Malaya, 50603 Kuala Lumpur, Malaysia; 2 Allergy and Immunology Research Centre, Institute for Medical Research, Jalan Pahang, 50588 Kuala Lumpur, Malaysia; 3 Centre of Research for Computational Sciences and Informatics for Biology, Bioindustry, Environment, Agriculture and Healthcare (CRYSTAL), University of Malaya, 50603 Kuala Lumpur, Malaysia

## Abstract

X-linked agammaglobulinemia (XLA) is a rare genetic disorder, caused by mutations in *BTK* (Bruton’s Tyrosine Kinase) gene. Deep high-throughput RNA sequencing (RNA-Seq) approach was utilized to explore the possible differences in transcriptome profiles of primary monocytes in XLA patients compared with healthy subjects. Our analysis revealed the differences in expression of 1,827 protein-coding genes, 95 annotated long non-coding RNAs (lncRNAs) and 20 novel lincRNAs between XLA patients and healthy subjects. GO and KEGG pathway analysis of differentially expressed (DE) protein-coding genes showed downregulation of several innate immune-related genes and upregulation of oxidative phosphorylation and apoptosis-related genes in XLA patients compared to the healthy subjects. Moreover, the functional prediction analysis of DE lncRNAs revealed their potential role in regulating the monocytes cell cycle and apoptosis in XLA patients. Our results suggested that *BTK* mutations may contribute to the dysregulation of innate immune system and increase susceptibility to apoptosis in monocytes of XLA patients. This study provides significant finding on the regulation of *BTK* gene in monocytes and the potential for development of innovative biomarkers and therapeutic monitoring strategies to increase the quality of life in XLA patients.

## Introduction

X-linked agammaglobulinemia (XLA) is one of the inherited forms of Primary Immunodeficiency Diseases (PIDs)^1^. It is caused by mutations in the *BTK* (Bruton’s Tyrosine Kinase) gene, which results in defective development and maturation of B cell within the bone marrow and a considerable decrease or complete absence of mature B cells in peripheral blood^2^. Due to the absence of mature B cells, XLA patients have significantly decreased levels of all major immunoglobulins in the serum and consequently, would be subjected to severe and chronic bacterial infections^3^. The *BTK* expression is not restricted to B cells, it is also expressed in myeloid cells such as neutrophils^4^, natural killer (NK) cells^5^, and monocytes^6^. The significance of *BTK* for macrophage function was first seen in X-linked immunodeficient (XID) mice infected with microfilaria^7^. The experiments showed a delayed microfilaria clearance together with low levels of *IL-12A* (Interleukin 12A), *IL-1* (Interleukin 1) and *TNF* (Tumor Necrosis Factor) production as well as decrease in *NO* (Nitric oxide) production in XID mice^7^. Similarly, Schmidt and colleagues showed that in primary macrophages, *BTK* was activated by *TLR4* (Toll-like Receptor 4) and is essential for normal *TLR*-induced *IL-10* (Interleukin 10) production in various populations of macrophages^8^. Additionally, *BTK* also plays a crucial role in initiating *TLR3* signaling in *BTK* deficient macrophages^9^. In the absence of *BTK*, *TLR3*-induced *PI3K* (Phosphoinositide 3-Kinase), *AKT* (V-Akt Murine Thymoma Viral Oncogene Homolog 1) and *MAPK* (MAP Kinase Phosphorylation) signaling as well as activation of *NFκB* (Nuclear Factor Kappa B), *IRF3* (Interferon Regulatory Factor 3), and AP-1 transcription factors were defective^9^. Further investigations on the human monocytic THP1 cell line showed interactions of *TLR8* and *TLR9* with *BTK*, in which defective *BTK* leads to impaired *TLR8* and *TLR9* signaling and causes susceptibility of XLA patients to viral infections^10^. It has also been reported that *BTK* contributed in *TLR4* signaling to *NFκB*, and may also involve in signaling by ligands for *TLR2*, *TLR*6, *TLR8*, and *TLR9* and also with *MYD88* (Myeloid Differentiation Primary Response 88), *MAL* (MyD88-Adapter-Like) and *IRAK1* (Interleukin 1 Receptor Associated Kinase 1)^11, 12^. The decreased chemotaxis and defective *FcγR* (Fc-gamma Receptors), *CR1* (Complement Receptor 1) and *CR3* (Complement Receptor 3)-mediated phagocytosis has also been reported in monocytes from XLA patients compared to healthy subjects^13^.

In addition to the protein coding genes, long non-coding RNAs (lncRNAs) have also been shown to play important roles in immune cell development and processes such as anti-viral responses, NFκB signaling, and inflammatory responses^14, 15^. lncRNAs are the biggest class of non-coding RNAs in mammalians, having more than 200 nucleotides length and without coding potential^16^. The lncRNAs dysregulated expression has been also reported in many human disease, such as cancer^17, 18^, neurological disorders^19^, autoimmune disease^20, 21^, and microbial susceptibility^22^.

Monocytes are essential components of the innate immune system. They are produced from a common myeloid progenitor cells in the bone marrow and circulate in the blood vessels for short times. During inflammatory conditions, they move into peripheral tissues, differentiating into macrophages and dendritic cells. The effect of primary monocyte with *BTK* deficient in XLA patients is not well studied. There is no or limited data exist on the genome-wide transcriptome expression profile of primary monocytes in XLA patients. In addition, the molecular mechanisms underlying the functions of lncRNAs in primary monocytes of XLA have not been studied yet. We recently published a gene reference catalogue and lncRNAs landscape of human primary monocytes from healthy subjects^23, 24^. In this study, we performed deep high-throughput RNA sequencing (RNA-Seq) analysis on primary monocytes from XLA patients and healthy subjects to investigate the effect of *BTK* gene expression deficiency on innate immune function of XLA patients. We identified the set of protein-coding genes and lncRNAs which were differentially expressed (DE) between XLA patients and healthy subjects. Gene Ontology (GO) and Kyoto Encyclopedia of Genes and Genomes (KEGG) pathway analyses predicted the functions of these DE protein-coding genes and lncRNAs in primary monocytes of XLA patients.

## Results

### Transcriptome profiling

We performed deep RNA-Seq of primary monocytes from healthy subjects and XLA patients. The patients were diagnosed to have XLA with absence or low circulating B cells, low serum immunoglobulin isotypes and deficient of BTK protein expression in their monocytes. Molecular genetic tests revealed *BTK* gene mutations in all patients. Novel *BTK* invariant splice site mutation was identified in one of the patients^25^. The history of the patient’s serum immunoglobulin levels before receiving intravenous human immunoglobulin (IVIG) therapy, and the nucleotide change that occurred in each patient and its consequences in the protein synthesis is shown in Table 1.

**Table 1:** Clinical and immunological data of the XLA patients.

					Ig levels at diagnosis (mg/dL)					Mutations		
Patient	Age (years)	Age at onset (years)^a^	Age at diagnosis (years)^b^	Family history^c^	IgG	IgM	IgA	CD19+ (%)	BTK expression	Nucleotide	Protein	Protein Domain
P1	12	1	4	−	N/A	N/A	N/A	1 (12−22)	7.7%	c.1888A > T	p.M630L	Kinase
P2	13	1	6	+	41(550−1200)	<12(40−95)	48 (60–170)	0 (12−22)	6%	IVS9 + 1 G > C	Skipping of exon 9	SH3 & SH2
P3	18	2	7	−	91.1 (550−1200)	11.3 (40−95)	UD (60−170)	0 (12−22)	0.04%	g.34430_34447 delCAAAGTCATGATgtgagt	p.A446_N451 ins(28 amino acids)	Kinase

N/A, not available. UD, undetectable. ^a^Age at the which an individual acquires, develops, or first experiences a condition or symptoms of a disease. ^b^Age at the start of intravenous immunoglobulin replacement. ^c^“ + ”, indicates that family members [boy (s)] died at a young age because of infection. ^d^Normal expression is >94%.

RNA was extracted from classical monocytes (CD14^++^CD16^−^) isolated from peripheral blood mononuclear cells (PBMCs) using a negative selection method. The purified RNA was used to carry out poly-adenylated paired-end RNA sequencing using Illumina HiSeq 2000 platform. After discarding adaptor sequences and low-quality sequences, we obtained approximately 1.2 million reads from all samples. The trimmed reads were mapped to human reference genome (Ensembl GRCH38.79) and transcripts were assembled using aligned reads. The transcripts from each XLA patients and healthy subjects were then separately merged to form two sets of single non-redundant transcripts. The expression levels of transcripts were quantified across each dataset. By applying an FPKM > 0.1 threshold, we have identified the expression of total 11,777 protein-coding genes and 3,363 lncRNAs in XLA patients, and 11,644 protein-coding genes and 3,190 lncRNAs in healthy subjects. Using multi-step mapping and filtering criteria, the expression of 430 and 380 potential novel lincRNAs were also identified in XLA patients and healthy subjects, respectively.

### Differentially expressed (DE) protein-coding genes and lncRNAs in XLA patients compared to healthy subjects

**Figure 1:**
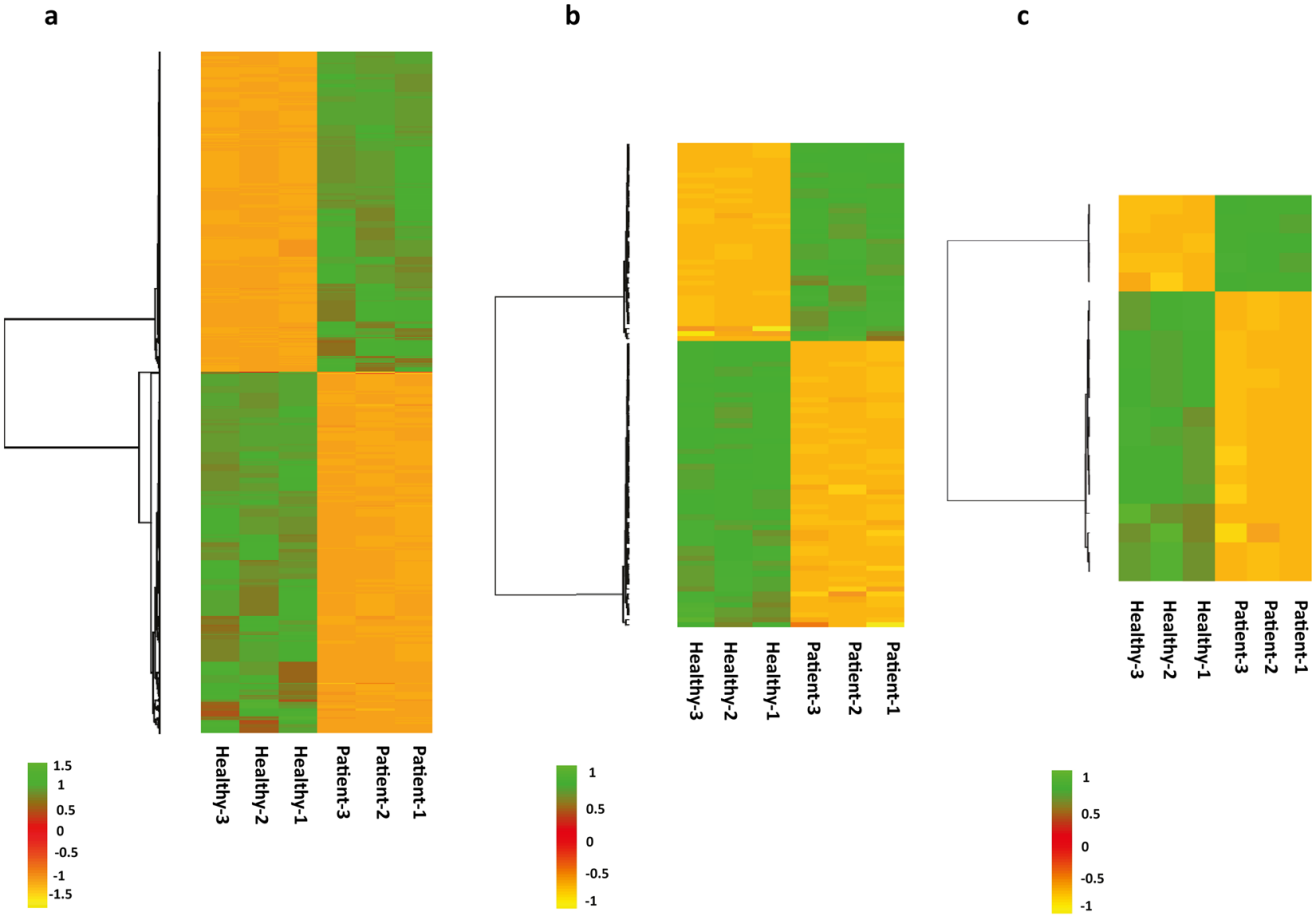
Hierarchical clustering of DE protein-coding genes and DE lncRNAs in primary monocytes of XLA patients compared to healthy subjects. (**a**) DE protein-coding genes (**b**) DE annotated lncRNAs and (**c**) DE novel lincRNAs. The green and orange shades indicate the expression above and below the relative expression, respectively, across all samples.

The differential gene expression analysis between XLA patients and healthy subjects was conducted using Cuffdiff and the genes with q-value ≤ 0.01 and log_2_ fold-change ≥ 1 or ≤ −1 were defined as differentially expressed (DE). Using these criteria, a total of 1,827 DE protein-coding genes, 95 DE annotated lncRNAs and 20 DE novel long intergenic non-coding RNAs (lincRNAs) were identified in XLA patients compared to the healthy subjects. Figure 1 shows the hierarchical clustering of the expression patterns of DE protein-coding genes and DE lncRNAs between XLA patients and healthy subjects.

### Differentially expressed (DE) protein-coding genes

**Figure 2:**
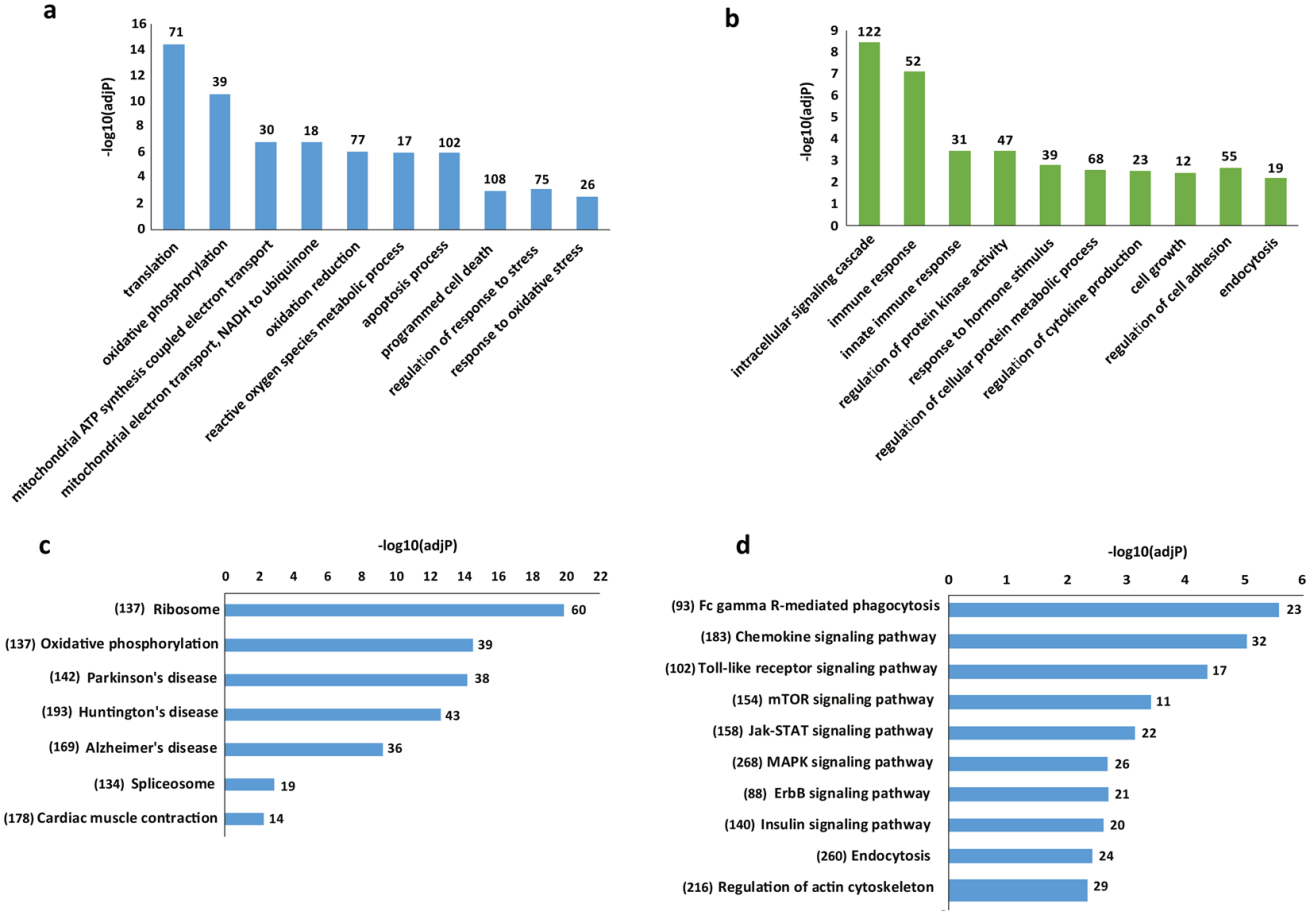
The GO and KEGG pathway analysis of DE protein-coding genes in primary monocytes of XLA patients compared to healthy subjects. The significant GO biological process terms (adj.P-value < 0.01) enriched for (**a**) upregulated genes, and (**b**) downregulated genes. The number of identified DE protein-coding genes enriched in each GO terms is depicted above bars of x-axis in the figure. The significant KEGG pathway terms enriched for (**c**) upregulated genes and (**d**) downregulated genes. Each statistical significance value (adj.p-value) was negative log-10 base transformed.The numbers in the brackets indicated the total number of genes available in the KEGG database for each pathway terms. The number of identified DE protein-coding genes enriched in each KEGG pathways is depicted above bars of x-axis in the figure.

Out of the 1,827 DE protein-coding genes, 859 genes were upregulated and 968 genes were downregulated in XLA patients compared to the healthy subjects (Supplementary Table S1). The expression of *BTK* was detected to be significantly downregulated (log_2_ fold-change < −7) in XLA patients compared to healthy subjects. The functional consequences of identified DE protein-coding genes in XLA patients compared to healthy subjects were characterized through GO enrichment and KEGG pathway analysis (Fig. 2, Supplementary Table S2). The significant GO biological process terms for upregulated genes were related to mitochondrial function and organization including: “oxidative phosphorylation”, mitochondrial ATP synthesis coupled to electron transport”, and “electron transport chain”, as well as “apoptotic” process and “response to oxidative stress” (Fig. 2a). The expression of several apoptosis-related genes such as *BAX* (BCL2 Associated X Protein) and *BAD* (BCL2 Associated Agonist of Cell Death) and oxidative stress response genes such as *SOD1* (Superoxide Dismutase 1, Soluble), *GPX1* (Glutathione Peroxidase 1), *GPX4* (Glutathione Peroxidase 4), *PRDX1* (Peroxiredoxin 1), *PRDX5* (Peroxiredoxin 5) were observed to be significantly upregulated in XLA patients compared to healthy subjects. However, the GO biological process terms for downregulated genes were significantly related to monocytes immune system functions including: “intracellular signaling cascade”, “immune response” and “innate immune response” (Fig. 2b). Furthermore, the KEGG pathway analysis revealed that the upregulated genes were enriched in 9 pathways, most significantly in “Oxidative phosphorylation” (Fig. 2c). The oxidative phosphorylation system consists of five protein complexes namely; I (*NDUF*; NADH: Ubiquinone Oxidoreductase), II (*SHD*; Src Homology 2 Domain Containing Transforming Protein D), III (*UQCR*; Ubiquinol-Cytochrome C Reductase), IV (*COX;* Cytochrome C Oxidase), and V (*ATP*; Adenosine Triphosphate). In this study, the upregulation of the several components of complexes I, III, IV and V were observed in primary monocytes of XLA patients compared to the healthy subjects (Table 2). The GO analysis also revealed that the downregulated genes were enriched in 29 pathways, most significantly in several immune-related pathways such as “Fc gamma R-mediated phagocytosis”, “Chemokine signaling pathway”, “Toll-like receptor signaling pathway” and “MTOR signaling pathway” (Fig. 2d). The core downregulated genes contributing to the enrichment of the immune-related pathways in primary monocyte of XLA patients is presented in Table 3.

**Table 2:** The upregulated genes involved in Oxidative Phosphorylation pathway in primary monocytes of the XLA patients compared to healthy subjects.

Oxidative Phosphorylation Pathway (P-value: 6.50E-18*;* Adj.P-value = 5.20E-16)	
Oxidative Phosphorylation system subunits	Gene Name
Complex I: NADH dehydrogenase	*NDUFA1*, *NDUFA12*, *NDUFA2*, *NDUFA3*, *NDUFA4*, *NDUFA6*, *NDUFA8, NDUFAB1, NDUFB11, NDUFB4, NDUFB5, NDUFB7, NDUFB8, NDUFS4, NDUFS5, NDUFS6, NDUFS7*
Complex III: Cytochorom c reductase	*UQCR10, UQCRB, UQCRFS1, UQCRH, UQCRHL*
Complex IV: Cytochorom c oxidase	*COX14, COX17, COX4I1, COX5A, COX5B, COX6B1, COX6C, COX7A2, COX7C*
Complex V: ATPase	*ATP1A3, ATP5D, ATP5E, ATP5G2, ATP5G3, ATP5H, ATP5I, ATP5J, ATP6V0B, ATP6V0E1, ATP6V1F*, *ATPIF1*

**Table 3:** The downregulated genes involved in immune-related pathways in primary monocytes of XLA patients compared to healthy subjects.

Pathway Terms	P-value	Adj.P-value	Gene Name
Fc gamma R-mediated phagocytosis	1.10E-07	2.60E-06	*FCGR2A, ASAP1, GAB2, DOCK2, DNM1L, INPP5D, MAPK1, MAP2K1, PAK1, PIKFYVE, PIK3CG, PIK3R5, PLCG1, PRKCB, PRKCE, PTPRC, SYK, LYN, VASP, VAV3, PIK3R1, RAF1, AKT2*
Chemokines signaling pathway	4.70E-07	9.30E-06	*CXCL16, CXCR4, CXCR1, CXCR2, JAK2, ROCK2, ADCY6, ADCY7, ADCY9, ADRBK2, DOCK2, FOXO3, GRB2, GNB4, GNG12, GNG2, MAPK1, MAP2K1, NRAS, PAK1, PIK3CG, PIK3R1, PIK3R5, PRKCB, PRKX, STAT2, STAT5B, ROCK1, SOS1, SOS2, BRAF, VAV3*
Toll-like receptors signaling pathway	3.10E-06	4.30E-05	*TLR1, TLR5, TLR2, TLR4, TLR6, TLR7, TBK1, IKBKE, JUN, MAPK1, MAP2K1, MAP2K4, MYD88, PIK3CG, PIK3R1, PIK3R5, FOS*
MTOR signaling pathway	5.20E-05	3.80E-04	*MTOR, RICTOR, HIF1A, MAPK1, PIK3CG, PIK3R1, PIK3R5, RPS6KA3, TSC1, ULK2, BRAF*
Jak-STAT signaling pathway	1.20E-04	7.10E-04	*CREBBP, CBL, EP300, JAK1, JAK2, CSF2RB, GRB2, IL10RA, IL13RA1, IL6R, IL6ST, PIK3CG, PIK3R1, PIK3R5, PRLR, STAT2, STAT5A, STAT5B, SOS1, SOS2, SOCS4, SOCS7*
MAPK signaling pathway	4.30E-04	2.10E-03	*RAPGEF2, ATF2, DUSP1, DUSP6, FLNB, GRB2, GNA12, GNG12, HSPA1A, JUN, MAPK1, MAP2K1, MAP2K4, MAP3K1, MAP4K4, NRAS, PAK1, PRKCB, PRKX, RPS6KA3, SOS1, SOS2, TGFBR1, FOS, BRAF*
ErbB signaling pathway	5.10E-04	2.40E-03	*CBL, EREG, GRB2, JUN, MTOR, MAPK1, MAP2K1, MAP2K4, NRAS, PAK1, PIK3CG, PIK3R, PIK3CG, PIK3R, PIK3R5, PLCG1, PRKCB, STAT5A, STAT5B, SOS1, SOS2, ABL2, BRAF*
Insulin signaling pathway	8.30E-04	3.80E-03	*CBL, GRB2, INPP5D, IRS2, MTOR, MAPK1, MAP2K1, NRAS, PDE3B, PIK3CG, PIK3R1, PIK3R5, PRKCI, PRKX, PTPRF, SOS1, SOS2, SOCS4, TSC1, BRAF*
Endocytosis	1.00E-03	4.50E-03	*ARAP2, ASAP1, ACAP2, CBL, DNAJC6, EHD3, IQSEC1, RAB11, FIP1, ADRBK2, CXCR4, CLTCL1, DNM1L, EPN2, HSPA1A, CXCR1, CXCR2, LDLR, NEDD4, PIKFYVE, PRKCI, RNF41, SMAP2, TGFBR1, VPS36*
Regulation of actin cytoskeleton	2.20E-03	9.60E-03	*IQGAP1, IQGAP2, ARHGEF12, ROCK2, ACTN1, CYFIP1, FGFR1, FN1, GNA12, GNA13, GNG12, ITGA4, ITGAV, ITGB1, MAPK1, MAP2K1, NRAS, PAK1, PIKFYVE, PIK3CG, PIK3R1, PIK3R5, PPP1R12A, ROCK1, SSH2, SOS1, SOS2, BRAF, VAV3*

### Gene interaction network of DE protein-coding genes

**Figure 3:**
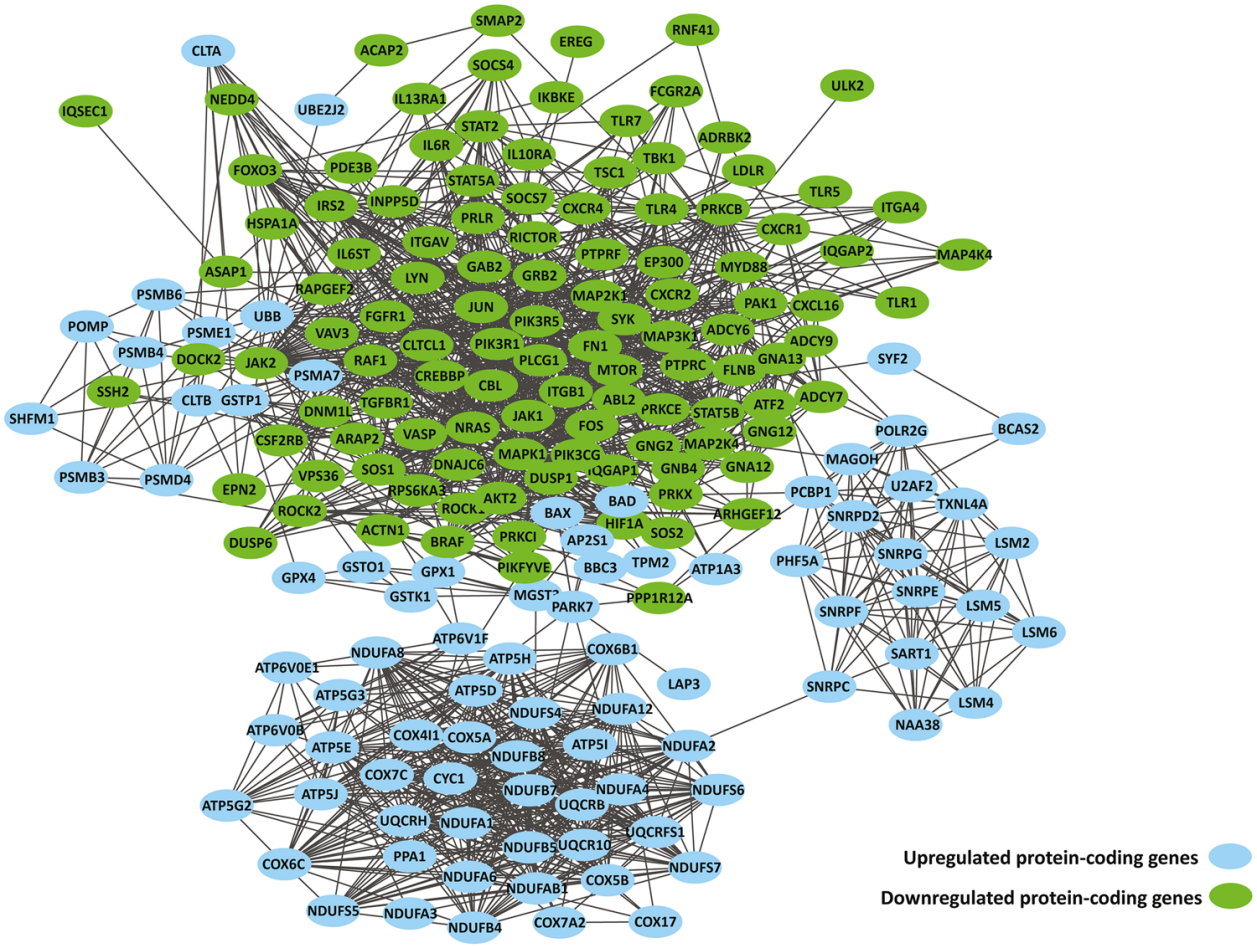
Interaction network analysis of DE protein-coding genes in primary monocytes of XLA patients compared to healthy subjects. The DE protein-coding genes were connected in a network based on the protein-protein interactions.

To explore the dysregulated gene interactions in primary monocytes of XLA patients, the interaction networks were generated for DE protein-coding genes which were significantly enriched in upregulated and downregulated KEGG pathways in XLA patients compared to healthy subjects (Fig. 3). The network contained 1,601 interactions between 78 upregulated and 103 downregulated genes.

### Differentially expressed (DE) long non-coding RNAs (lncRNAs)

The total of 95 DE annotated lncRNAs were detected in primary monocytes of XLA patients compared to healthy subjects in which 56 and 39 lncRNAs were upregulated and downregulated, respectively (Supplementary Table S3). Several lncRNAs which were known to be involved in the regulation of gene expression, cell cycle and apoptosis were detected among DE lncRNAs in primary monocytes of XLA patients. These lncRNAs include: *HOTAIRM1* (HOXA Transcript Antisense RNA, Myeloid-Specific 1)^26^, *DANCR* (Differentiation Antagonizing Non-Protein Coding RNA)^27^, *GAS5* (Growth Arrest Specific 5)^28^, *LINC-PINT* (Long Intergenic Non-Protein Coding RNA, P53 Induced Transcript)^29^, *HEIH* (Hepatocellular Carcinoma Associated Transcript)^30^ and *RMRP* (RNA Component Of Mitochondrial RNA Processing Endoribonuclease)^31^ which were upregulated and, *TUG1* (Taurine Upregulated 1)^32^, which was downregulated in XLA patients compared to the healthy subjects. In addition, expression of the 20 DE novel lincRNAs were identified between XLA patients and healthy subjects. Among the 20 DE novel lincRNAs, 5 lincRNAs were upregulated and 15 lincRNAs were downregulated in XLA patients compared to healthy subjects, respectively (Supplementary Table S4).

### DE lncRNAs co-located with protein-coding genes

lncRNAs have been reported to coordinate the regulation of neighboring protein-coding genes (co-located genes). To identify the potential function of the identified DE lncRNAs, the protein-coding genes which genomic locations within ~5 kb upstream and ~1 kb downstream of the DE lncRNAs and which may extend to 1000 kb in both directions were searched. The analysis revealed that out of 95 DE annotated lncRNAs, 85 lncRNAs were corresponded to 144 protein-coding genes. Moreover, among the 20 DE novel lincRNAs, 18 novel lincRNAs were linked to 28 protein-coding genes (Supplementary Table S5). Majority of DE lncRNAs region-gene associations were found to be distal binding events while approximately only 9% regions were within 5 kb of transcription start sites (TSS) (Supplementary Fig. S1).

**Figure 4:**
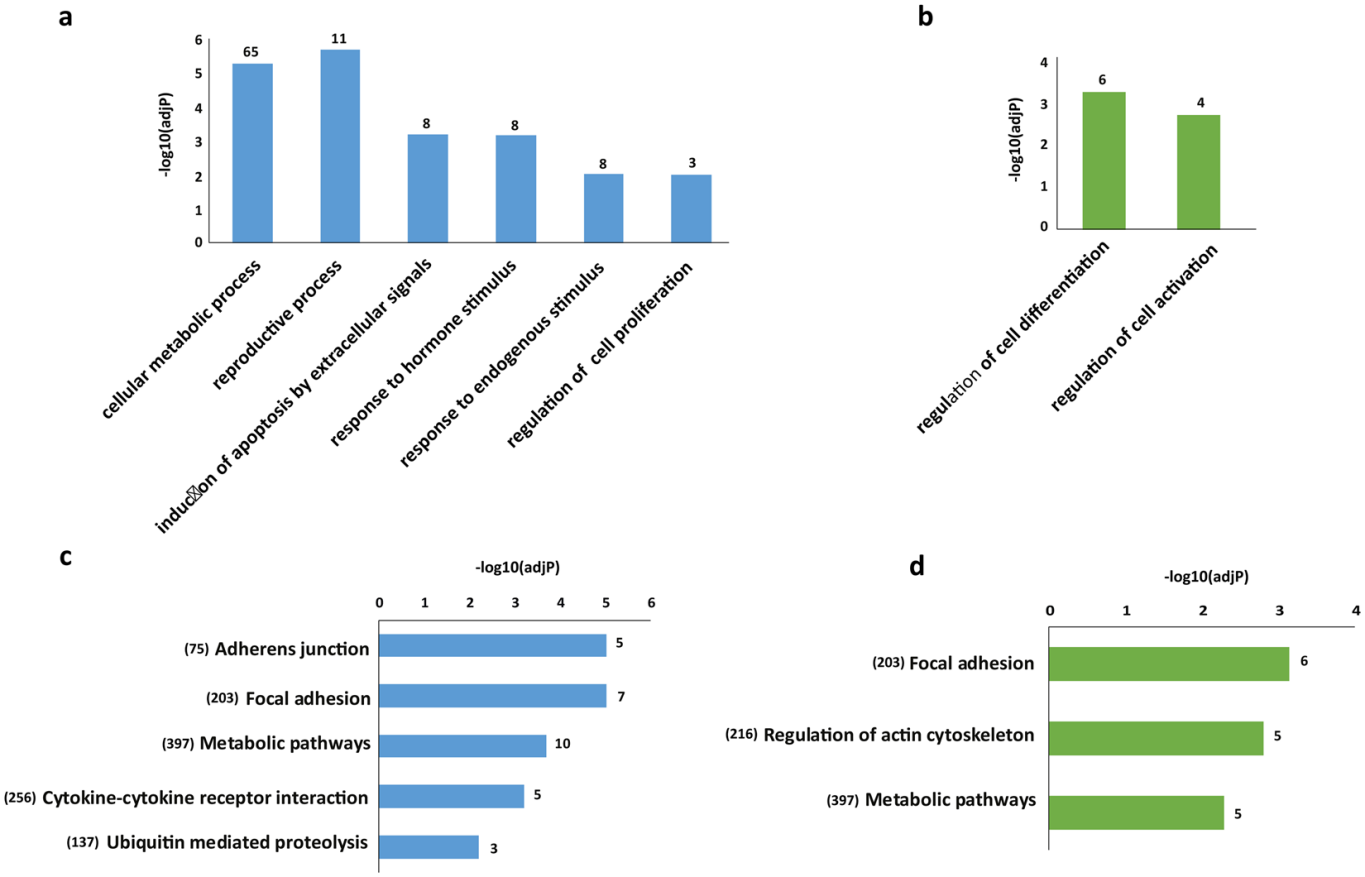
The GO and KEGG pathway analysis of DE lncRNAs co-located genes in primary monocytes of XLA patients compared to healthy subjects. The significant GO biological process terms enriched for (**a**) DE annotated lncRNAs co-located genes, and (**b**) DE novel lincRNAs co-located genes. The number of DE lncRNAs co-located genes enriched in each GO terms is depicted above the bars in the figure. The significant KEGG pathway terms enriched for (**c**) DE annotated lncRNAs co-located genes, and (**d**) DE novel lincRNAs co-located genes. Each statistical significance value (adj.p-value) was negative log-10 base transformed. The numbers in the brackets indicate the total numbers of genes available in the KEGG database for each pathway terms. The number of identified DE lncRNAs co-located genes enriched in each KEGG pathways is depicted above the bars in the figure.

Next, the function of DE lncRNAs was examined based on GO enrichment and KEGG pathway analysis of their co-located genes (Fig. 4, Supplementary Table S2). The GO enrichment analysis based on biological process demonstrated that the DE annotated lncRNAs co-located genes were mainly involved in “reproductive process”, “positive regulation of cell proliferation” and “induction of apoptosis by extracellular signals” (Fig. 4a). On the other hands, the DE novel lincRNAs co-located genes were related to “regulation of cell differentiation” and “regulation of cell activation” (Fig. 4b). The KEGG pathway analysis revealed that the DE annotated lncRNAs co-located genes were significantly involved in “Metabolic pathways”, and “Cytokine- cytokine receptor interaction” (Fig. 4c). While the novel lincRNAs co-located genes were related to “Focal adhesion” and “Regulation of actin cytoskeleton” (Fig. 4d).

Besides that, we examined whether any DE lncRNAs co-located genes was differentially expressed in XLA patients compared to healthy subjects. The comparison of the DE annotated and novel lncRNAs co-located genes with DE protein-coding genes led to identification of 23 genomically co-located DE protein-coding genes (Supplementary Table S6). For instance, DE annotated lncRNAs; *HOTAIRM1*, *DANCR and GAS5* were co-located with DE protein-coding genes: *HOXA1*, *USP46*, *ZBTB48* respectively, which are involved in regulating the gene expression, morphogenesis and differentiation, ubiquitin protease activity, and MHC II promoter compotes. The results also indicated that DE novel lincRNAs *TCONS_00030433*, *TCONS_00041961* and *TCONS_00295657* were co-located with DE protein-coding genes *VAV3* (Vav Guanine Nucleotide Exchange Factor 3) - involve in phagocytosis, *DOCK1* (Dedicator of cytokinesis) - involve in kinase activity and *DUSP22* (Dual Specificity Phosphatase 22) - involve in ubiquitin protease activity.

### Gene interaction network of DE lncRNAs with their co-located DE protein-coding genes

**Figure 5:**
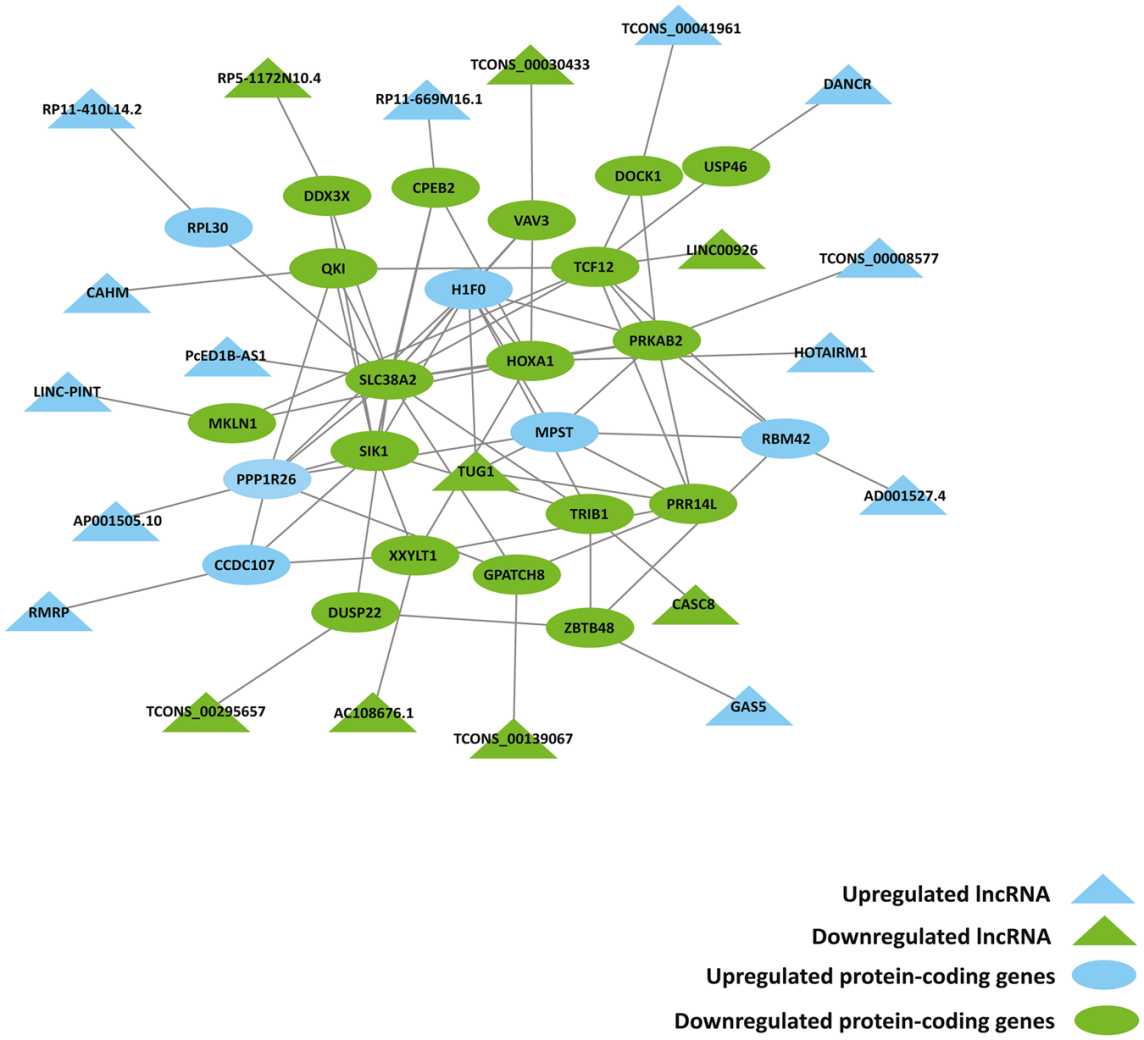
The interaction network of DE lncRNAs with their co-located DE protein-coding genes in primary monocytes of XLA patients compared to healthy subjects. The DE protein-coding genes were connected in a network based on the protein-protein interactions. The edges for lncRNAs that were close in genomic space to the DE protein-coding genes were automatically added into the network.

To unravel the interaction between DE annotated lncRNAs and DE novel lincRNAs and their co-located DE protein-coding genes, putative interactive networks were constructed using Cytoscape. The network contained 80 interactions between 21 DE annotated and DE novel lincRNAs with 23 co-located DE protein-coding genes (Fig. 5).

### qRT-PCR validation

**Figure 6:**
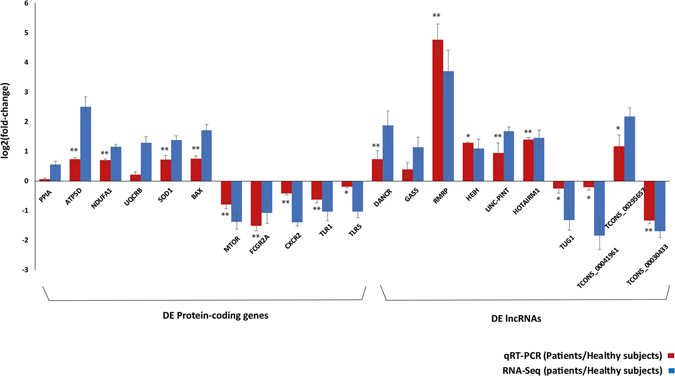
The qRT-PCR validation of DE protein-coding genes and DE lncRNAs in primary monocytes of XLA patients compared to the healthy subjects. The comparison of log_2_ fold-change of DE protein-coding genes and DE lncRNAs were determined by RNA-Seq analysis (blue) and qRT-PCR validation (red). *PPIA* was used as endogenous control for normalizing the expression levels. x-axis shows genes; y-axis shows the log_2_ ratio of expression in XLA patients compared to healthy subjects. Statistical significance was calculated using paired Student’s T-test. The asterisks above the bars denote statistically significant differences from healthy subjects obtained by qRT-PCR, *P-value < 0.05, **P-value < 0.01.

To further confirm our findings as described above, expression levels of selected DE protein-coding genes and DE annotated and novel lincRNAs were measured by Quantitative Reverse Transcription Polymerase Chain Reaction (qRT-PCR) analysis. The candidate genes were 10 DE protein-coding genes: *FCGR2A*, *CXCR2*, *TLR1*, *TLR5*, *ATP5D*, *NDUFA1*, *UQCRB*, *SOD1*, *MTOR* and *BAX* which were enriched in several significant upregulated and downregulated KEGG pathways. Also included were 7 DE annotated lncRNAs; *HOTAIRM1*, *DANCR*, *GAS5*, *LINC-PINT*, *RMRP*, *HEIH*, *TUG1* and 3 DE novel lincRNAs; *TCONS_00041961*, *TCONS_00295657*, *TCONS_00298577*, which co-located with DE protein-coding genes in XLA patients compared to the healthy subjects. The qRT-PCR validation results showed that in total, 90% of the selected genes reached significance (P-value ≤ 0.05) (Fig. 6). Comparison between RNA-Seq and qRT-PCR log_2_ fold-change ratio revealed similar expression trends for selected genes with a Pearson correlation (r) value of 0.83 (P-value < 0.001), demonstrating the reliability of our RNA-Seq data analysis (Fig. 6).

## Discussion

In this study, we investigated the effect of *BTK* gene expression deficiency on primary monocyte’s immune function in XLA patients using deep RNA-Seq analysis. The *BTK* gene is situated at band Xq21.3 to Xq22, long arm of the X chromosome, spanning 37.5 kb that contains 19 exons. The BTK protein has 5 different functional domains including *PH*, *TH*, *SH3*, *SH2*, and *TK*. Over 800 mutations have been identified in *BTK* ^33^. These mutations varied in types and scattered throughout all domains of the BTK protein, which all resulting in overall dysfunction of BTK protein. Recently, Xia-Fang and colleagues examined the genetic background and clinical features of 174 patients with XLA and reported that there was no relationship between *BTK* mutations and clinical symptoms in XLA patients^34^.

We compared transcriptome profiles of primary monocytes of healthy subjects and XLA patients, who shared similar clinical symptoms despites having different type of mutations in *BTK* gene. Our analysis showed a total of 1,827 DE protein-coding genes, of which 859 genes were upregulated and 968 genes were downregulated in XLA patients compared to the healthy subjects (Supplementary Table S1). Based on the GO and KEGG pathways analysis, detailed information on the biological functions and potential mechanisms of actions of DE protein-coding genes were identified. The GO enrichment analysis showed that downregulated genes were mainly involved in the regulation of immune response. Pathway enrichment analysis also revealed that downregulated genes were mainly enriched in several pathways belonged to the innate immune system such as: “Fc gamma R-mediated phagocytosis”, “Chemokine signaling pathways”, “Toll like receptors signaling pathway” and “MTOR signaling pathway”, reflects the deficiencies of innate immune function in primary monocytes of the XLA patients.

The expression of *FCGR2A* (also known as FcγRIIA or CD32, involved in “Fc gamma R-mediated phagocytosis”) was significantly decreased in primary monocytes of the XLA patients which is consistent with the findings about decreased expression of *FCGR2A* in monocytes from XLA patients due to BTK deficiency^13^. In addition to *FCGR2A*, our study identified the downregulation of 22 core enrichment genes involved in “Fc gamma R-mediated phagocytosis” pathway in XLA patients compared to healthy subjects (Table 3). Majority of these genes encode for kinases in the early signaling events [such as *LYN* (LYN Proto-Oncogene, Src Family Tyrosine Kinase) and *SYK* (Spleen Tyrosine Kinase) Kinases] as well as the genes encode for proteins involved in cytoskeleton rearrangement. Decreased expression of 32 core genes involved in “chemokines signaling pathway” including 4 chemokine receptors (*CXCL16*, *CXCR1*, *CXCR2*, *CXCR4*) were also observed in primary monocytes of the XLA patients (Table 3). A direct role for *BTK* in signaling by *CXCR4* and in chemokine-controlled adhesion and migration in B cells of XLA patients has been shown previously^35^. Similar observations regarding the regulatory role of *BTK* on *CXCR4* as well as *CXCL16*, *CXCR1*, and *CXCR2* were found in primary monocytes of XLA patients in which the *BTK* deficiency may lead to downregulation of these chemokine receptors.

Another significant finding of this study is the overall downregulation of “Toll-like Receptor (TLR) signaling pathway” in primary monocytes of the XLA patients compared to healthy subjects (Table 3). The *BTK* has been shown to be involved in *TLR* signaling, where it interacted with *TLR2*, *TLR4*, *TLR6, TLR7*, *TLR8* and *TLR9* and facilitated their transduction of downstream signals and phosphorylation^10–12^. In addition to these findings, our study demonstrated for the first time, significant decreased in expression of *TLR1* and *TLR5* in primary monocytes of the XLA patients compared to healthy subjects. This suggested that the *BTK* may also associates with *TLR1* and *TLR5* expression in primary monocyte of XLA patients, in which mutants of *BTK* may inhibit their signaling. Similar observation of *TLR1* signaling deficiency due to the *BTK* mutant was reported in mice^36^.

Our analysis also revealed the downregulation of the *MTOR* (Mechanistic Target Of Rapamycin) genes along with the other 10 core genes of the “MTOR signaling pathway” in the XLA patients compared to healthy subjects (Table 3). Recently, Ezell and colleagues reported similar observation regarding the possible regulatory role of *BTK* on *MTOR* signaling in activated Diffuse Large B Cell Lymphoma (DLBCL)^37^. *MTOR* plays an important role in cell differentiation and growth, cellular metabolism and cancer metabolism. It can sense the growth factors, nutrients, insulin, energy and environmental changes, then transmit signals to downstream targets to activate the metabolic and cellular reactions^38^. In addition, *MTOR* was recently found to be associated with the regulation of both the innate^39^ and adaptive immune response^40^. Low expression of *MTOR* was observed in cells that are more dependent on mitochondrial oxidative phosphorylation for energy supply^41^. Overexpression of mitochondrial components due to great energy production demand has been reported in several disease states such as cancers^42^, Acquired Immune Deficiency Syndrome (AIDs)^43^ and Alzheimer’s disease (AD)^44^. We also detected low expression of *MTOR* and overexpression of multiple components of mitochondrial complexes I (*NDUF*; NADH: Ubiquinone Oxidoreductase), III (*UQCR*; Ubiquinol-Cytochrome C Reductase), IV (*COX;* Cytochrome C Oxidase) and V (*ATP*; Adenosine Triphosphate) in XLA patients compared to the healthy subjects (Table 2). This indicate great energy demand in primary monocytes of the XLA patients compared to healthy subjects. Furthermore, the upregulated genes involved in the production of reactive oxygen species (ROS), oxidative stress response and apoptotic process were observed in the XLA patients compared to healthy subjects. It is well known that during the oxidative phosphorylation, mitochondria consume most of the cellular oxygen and produce the majority of ROS. High concentration of ROS in the cell would lead to state termed oxidative stress, in which the excess ROS induces oxidative damage on cellular components and activate apoptosis pathways and cell death^45^. This may possibly explain the upregulation of several genes involved in the oxidative phosphorylation, ROS production, response to oxidative stress and apoptosis in monocytes of XLA patients.

In addition to the protein-coding genes, lncRNAs can also act as key regulators of various biological processes in the immune system^16, 17^. Our analysis of the lncRNAs expression between the XLA patients and healthy subjects showed a total of 95 DE annotated lncRNAs (56 upregulated and 39 downregulated) and 20 DE novel lincRNAs (5 upregulated and 15 downregulated) (Supplementary Tables S3 and S4). Several DE lncRNAs were identified in the XLA patients, which were known to contribute to regulation of gene expressions and cell cycle. Overexpression of these lncRNAs have been reported to suppress the cell growth, differentiation, proliferation, and apoptosis in various diseases. Such lncRNAs include: *HOTAIRM1* ^26^, *DANCR* ^27^, *GAS5* ^28^, *LINC-PINT* ^29^, *HEIH* ^30^, *RMRP* ^31^, which have found to be overexpressed in primary monocytes of the XLA patients compared to the healthy subjects. By comparing to healthy subjects, our analysis also detected significant decreased in expression of lncRNA *TUG1* in the XLA patients. Downregulation of *TUG1* has been reported to inhibit osteosarcoma cell proliferation and promote apoptosis^32^. Similar observation regarding the dysregulated expression of these lncRNAs seen in the primary monocytes of XLA patients, suggesting their possible role in regulating monocyte cell cycle and apoptosis in XLA patients. The analysis of DE novel lincRNAs also revealed that some DE novel lincRNAs were co-located with DE protein-coding genes related to immune system. In particular, the novel DE lncRNA *TCONS_00030433*, which was significantly downregulated in the XLA patients, interacted with *VAV3*, of which its expression was also detected to be downregulated in the XLA patients. *VAV3* is known to be involved in “Fc gamma R-mediated phagocytosis” pathway^46^. These results would suggest the possible role of *TCONS_00030433* in the regulation of “Fc gamma R-mediated phagocytosis” pathway in primary monocytes of XLA patients.

In summary, our analysis based on deep RNA-Seq datasets revealed that the expression profiles of protein-coding genes were significantly altered in primary monocytes of XLA patients compared to the healthy subjects. Regardless type of mutations, loss of function mutation in *BTK* genes eventually induced dysfunction of *BTK* protein, block the development of B-cell and caused the disease. The functional analysis of differentially expressed (DE) protein-coding genes showed that the impaired immune function and increased susceptibility to apoptosis in monocytes of XLA patients were due to *BTK* deficiency. This showed that *BTK* is not only involved in the development and function of B cells, but it may play an important role in establishing the immunity function of monocytes. Our study also revealed the differential expression patterns of lncRNAs in primary monocytes of XLA patients compared to healthy subjects and predicted their potential role in regulation of monocyte cell cycle and apoptosis in XLA patients. However, the specific biological functions and molecular mechanisms of these lncRNAs in monocytes of XLA patients required additional evaluation. Moreover, further large scale studies utilising integrated omics approaches would advance our knowledge and understanding on the impaired immune function of monocytes in XLA patients.

## Methods

### Sample collection and purification

Ethics approval to conduct this study was obtained from Medical Research and Ethics Committee (MREC), Malaysia with reference number NMRR-13-972-16921. Three unrelated male patients with XLA disease and 3 unrelated healthy male subjects were selected in this study. The written informed consent from all subjects for the use of their blood samples were obtained. The blood samples were handled according to the guidelines of the Helsinki Declaration. The entire experiment was conducted in accordance with the guidelines of Medical Research and Ethics Committee (MREC), Malaysia. The healthy subjects were non-smokers, did not have any medical illness, did not prescribed with any long-term medication and did not receive any vaccination at least 6 months before the study. The patients were diagnosed to have XLA disease based on the criteria of the World Health Organization Scientific Group for PIDs: low levels of circulating B cells (measured by levels of CD19^+^ B cells in blood samples), reduced or absent of immunoglobulins in serum and a typical clinical history with recurrent bacterial infection or a positive family history^47^. All selected patients had deficient monocyte BTK expression, as evaluated by flow cytometry. *BTK* gene mutations in all patients were confirmed by Sanger sequencing. All the patients were in a stable clinical situation without fever and not hospitalized when the blood was taken. They were under monthly IVIG therapy. The blood samples from patients were collected before the administration of IVIG. The 10 mL of peripheral blood from each healthy subjects and XLA patients were collected in EDTA tubes. Peripheral blood mononuclear cells (PBMCs) were isolated using Ficoll-based density gradient centrifugation. The classical monocytes (CD14^++^CD16^−^) were isolated from PBMCs by negative selection method using monocyte isolation kit II (Miltenyi Biotec, Germany). The BD FACSCanto II Flow Cytometer (BD Biosciences, USA) was used to perform the purity check of the isolated monocytes.

### RNA extraction, library preparation and sequencing

Total RNA was extracted from monocytes using RNeasy mini-kit followed by DNase treatment (QIAGEN, Germany). The quality and quantity of RNA was measured using NanoDrop 2000 (Thermo Fisher Scientific Inc, USA) and Qubit 2.0 RNA Broad Range Assay (Invitrogen, USA). The RNA purity was checked using Agilent Bioanalyzer RNA Nano chip Bioanalyzer (Agilent Technologies, USA). All samples had RNA Integrity Number (RIN) higher than 9. Messenger RNA isolation and cDNA synthesis were performed using TruSeq RNA Sample Preparation Kit (Illumina, USA) and Super Script II Reverse Transcriptase (Invitrogen, USA). The synthesized cDNA was quantified using Qubit 2.0 DNA Broad Range Assay (Invitrogen, USA). A minimum of 40ng cDNA was fragmented using Covaris S220 (Covaris INC, USA) to a targeted size of 200–300 bp. The fragmented cDNA was then end-repaired, ligated to Illumina TruSeq adaptors, and PCR-enriched using TruSeq RNA sample preparation kit (Illumina, USA). The final sequencing libraries were quantified using KAPA kit (KAPA Biosystem, USA) on Agilent Stragene Mx-3005p quantitative PCR (Agilent, USA) and sizes were confirmed using Agilent Bioanalyzer High Sensitivity DNA Chip (Agilent, USA). The RNA libraries were sequenced on Illumina HiSeq 2000 Platform (Illumina, USA) to generate 2 × 100 bp paired-end sequencing reads. The quality of sequences in fastq format was evaluated using FASTQC^48^. Low quality bases and adaptors were trimmed from the sequences using Trimmomatic^49^.

### Alignment and transcript assembly

Quality trimmed sequences from all samples were separately mapped to the human reference genome sequence (Ensemble GRCh38.79) using HISAT (version 0.1.4)^50^ with GENCODE junctions as a guided reference annotation and the transcript assembly was performed with StringTie (version 1.3.3)^51^ using a GENCODE reference annotation (version 22). The transcripts abundance was estimated as fragments per kilobases of exon per million fragments mapped (FPKM)^52^. The assembled transcripts of XLA patients and healthy subject were separately merged by using Cuffmerge (a part of cufflinks, version 2.2.1)^52^. The merged assembled files were then compared with a GENCODE reference annotation file (version 22) to obtain the number of expressed protein-coding genes and lncRNAs in the XLA patients and healthy subjects.

### Identification of novel long intergenic non-coding RNAs (lincRNAs)

Using an established pipeline for identification of lncRNA^24^, we analyzed the expression of putative novel long intergenic non-coding RNAs **(**lincRNAs) in primary monocytes. Briefly, transcripts with coverage of 3 x and above were filtered from the patients and healthy subjects’ transcripts assembled files. Then the filtered transcript files of each dataset were separately merged to form a non-redundant set of transcripts using Cuffmerge. The expression level of each transcript was quantified using Cuffquant (a part of Cufflinks, version 2.2.1)^52^ and normalized for total number of reads using Cuffnorm (a part of Cufflinks, verstion 2.2.1)^52^. The transcripts were mapped to the known gene annotation from GENCODE (version 22) and categorized as protein-coding, lncRNAs, novel and others (such as tRNAs, microRNAs, pseudogenes, etc). All known transcripts were filtered out while novel transcripts were retained. Only novel transcripts with more than 200 nucleotides which are intergenic to any GENCODE transcript were considered for further analysis. The coding potential of transcripts was then evaluated using Coding Potential Assessment Tool (CPAT)^53^. Coding probability cut-off of 0.375 was used to mark any putative protein coding sequences and the sequences were excluded from our analysis. The nucleotide sequences of putative novel lincRNAs were searched for any matching sequences against the non-redundant (NR) database from NCBI using BLASTN and no significant homology was found.

### Differential gene expression analysis

Differential gene expression analysis was performed by employing Cuffdiff (a part of Cufflinks, version 2.2.1)^52^. The DE genes were identified with q-value ≤ 0.01 and log_2_ fold-change ≥ 1 or ≤ −1. Hierarchical clustering of the expression profiles of detected DE protein-coding genes and DE lncRNAs were done with the heatmap function in the R “NMF” package^54^ using Pearson Correlation.

### GO and KEGG pathway analysis of DE protein-coding genes

To investigate the biological importance of the identified DE protein-coding genes, DAVID (Database for Annotation, Visualization and Integrated Discovery) functional annotation tool^55^ was used to perform Gene Ontology (GO) analysis^56^. DAVID uses modified Fisher’s exact test (known as EASE)^57^ to measure enrichment against a background gene list and adjusting the resulting p-values (adj.P-value) using a Benjamini-Hochberg method^58^. The GO analysis was restricted to the category of biological processes as it is the most prominent for evaluation of genes function. The DAVID category-GOTERM_BP_FAT level was selected for displaying the results. The minimum number of genes for enrichment in each category was set at 2 and the significance cutoff was adj.P-value < 0.01. Subsequently, the pathway analysis of identified protein-coding genes was conducted by applying the Kyoto Encyclopedia of Genes and Genomes (KEGG) database^59^ in DAVID tool using the same parameters.

### Co-location analysis DE lncRNAs with protein-coding genes

lncRNAs are presumed to regulate the expression of their neighboring genes (co-located genes)^60^. To predict the function of DE lncRNAs through their co-located genes, the genomic coordinates of DE lncRNAs were imported to Genomic Regions Enrichment of Annotations Tool (GREAT)^61^. The GO and KEGG pathway analysis of DE lncRNAs co-located genes was performed using the DAVID functional annotation tool.

The identified co-located genes were then matched with DE protein-coding genes to obtain a lncRNAs co-located genes which were also differentially expressed between XLA patients and healthy subjects^62^.

### Gene interaction network construction

In order to predict the DE protein-coding genes interaction, the protein-protein interaction network was constructed using Cytoscape plug-in GeneMANIA^63^ for genes which were significantly enriched (adj.P-value < 0.01) in upregulated and downregulated KEGG pathways. In addition, gene interaction networks between two subgroups of genes (DE lncRNA and their co-located DE protein-coding genes) were also constructed using Cytoscape^64^. The possible protein-protein interactions between DE protein-coding genes in each subgroup was predicted using the Cytoscape plug-in GeneMANIA. The network edges automatically added for lncRNAs that were close in genomic space to the DE protein-coding genes.

### Validation of RNA-Seq results by qRT-PCR

The expression levels of selected DE protein-coding and DE lncRNAs were validated using Quantitative Real-Time Polymerase Chain Reaction (qRT-PCR) analysis. First-strand cDNA was synthesized from 300 ng of RNA from each sample by using the High Capacity RNA to cDNA Kits (Applied Biosystems, USA). All the primers and probes for Taqman® Real-time PCR (Life Technologies, USA) were designed by Applied Biosystems (Supplementary Table S7). Expression of target genes was assessed using the QuantStudio™ 12 K Flex Real-Time PCR System. The conditions of the PCR cycle conditions were: 50 °C for 2 minutes, 95 °C for 20 seconds, followed by 40 cycles of 95 °C for 3 seconds, and 40 cycles of 60 °C for 30 seconds. Each gene was analyzed in triplicates for each sample. *PPIA* (Peptidylprolyl Isomerase A) gene was used as an endogenous control. Fold-changes in gene expression between samples were calculated using 2^−ΔΔCt^ method^65^. The Student’s t-test was used to evaluate the expression differences of selected protein-coding genes and lncRNAs between XLA patients and healthy subjects. P-value < 0.05 was considered as statistically significance.

### Availability of data and materials

The data discussed in this publication have been deposited in NCBI’s Gene Expression Omnibus (GEO) and are accessible through GEO series accessions number GSE80095 and GSE89980.

## Electronic supplementary material

Supplementary Figure S1

Supplementary Table S1

Supplementary Table S2

Supplementary Table S3

Supplementary Table S4

Supplementary Table S5

Supplementary Table S6

Supplementary Table S7
