# Supplementary material for: Transcriptome profiling of monocytes from XLA patients revealed the innate immune function dysregulation due to the BTK gene expression deficiency
Source: Sci Rep. 2017 Jul 28;7:6836. doi: 10.1038/s41598-017-06342-5 (PMC5533715; doi:10.1038/s41598-017-06342-5)
Supplement: Supplementary file 1 — Supplementary Figure S1 [file 41598_2017_6342_MOESM1_ESM.pdf]

Supplementary Figure S1

Transcriptome profiling of monocytes from XLA patients revealed the innate immune function dysregulation due to the BTK gene expression deficiency.

Hoda Mirsafian, Adiratna Mat Ripen, Wai-Mun Leong, Chai Teng Chear, Saharuddin Bin Mohamad, Amir Feisal Merican.

* Corresponding author:

Amir Feisal Merican

Institute of Biological Sciences, Faculty of Science, University of Malaya, 50603 Kuala Lumpur, Malaysia.

Email: merican@um.edu.my, mericanmy@gmail.com

Telephone: +60379674189

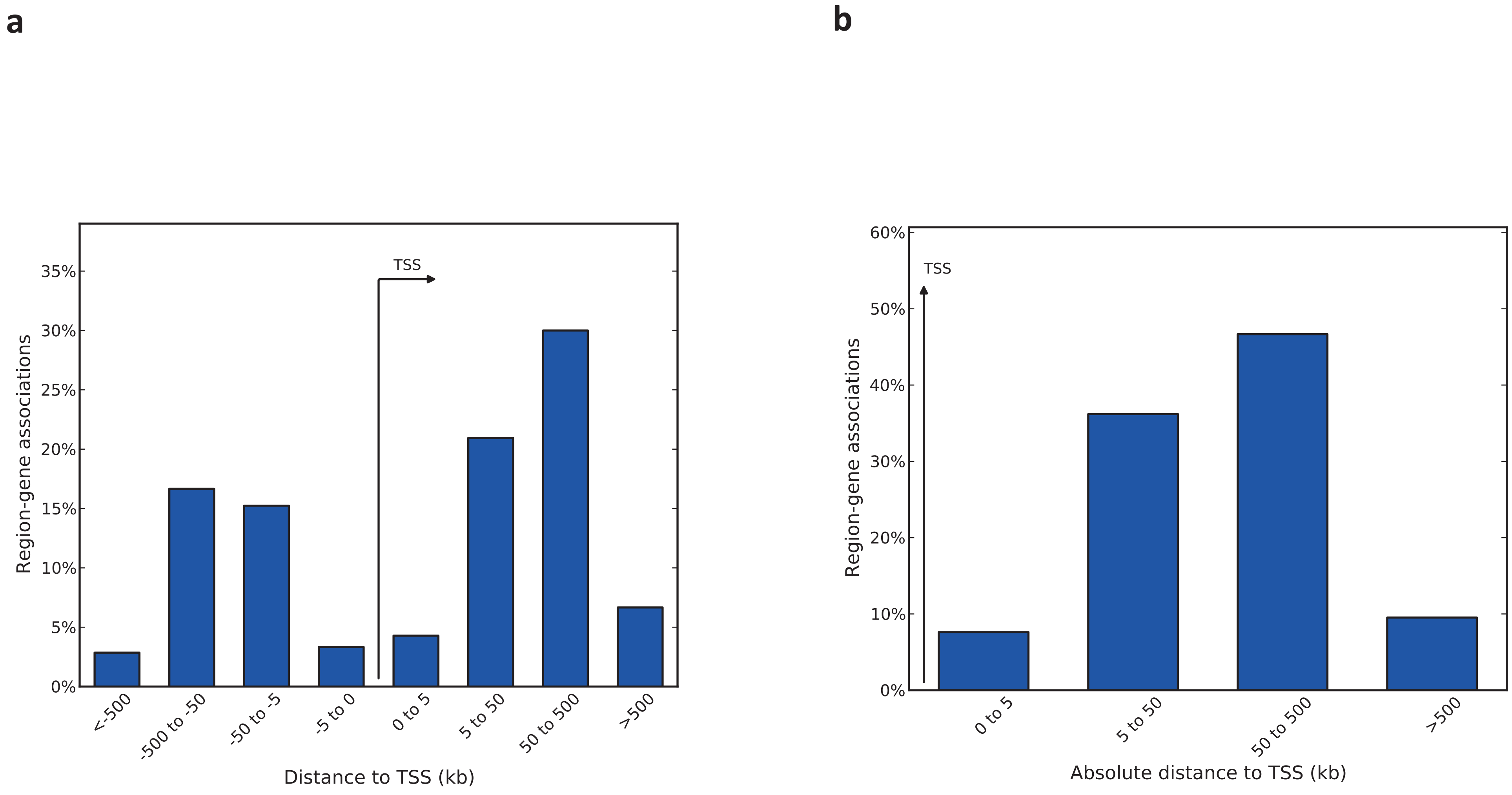
a
b
35%
30%
25%
20%
15%
10%
5%
0%
TSS
Region-gene associations
<-500
-500 to -50
-50 to -5
-5 to 0
0 to 5
5 to 50
50 to 500
>500
Distance to TSS (kb)
60%
50%
40%
30%
20%
10%
0%
TSS
Region-gene associations
0 to 5
5 to 50
50 to 500
>500
Absolute distance to TSS (kb)
